# Prevalence of Common Gastrointestinal Parasites in Small Ruminants and Their Associated Risk Factors in Farta District, Northwest Ethiopia

**DOI:** 10.1155/vmi/5465486

**Published:** 2026-11-03

**Authors:** Balemual Abebaw

**Affiliations:** ^1^ Department of Veterinary Clinical Medicine School of Veterinary Medicine Bahir Dar University Bahir Dar Ethiopia bdu.edu.et

**Keywords:** Farta District, gastrointestinal tract parasites, prevalence, risk factor, small ruminants

## Abstract

**Background:**

Infections caused by parasites in the gastrointestinal tract pose a significant health problem, negatively impacting the productivity and reproductive abilities of sheep and goats in Ethiopia.

**Materials and Methods:**

A cross‐sectional study was carried out in Farta District, located in the South Gondar Zone of Northwest Ethiopia, between October 2024 and August 2025, to estimate the prevalence of common gastrointestinal parasite infections in small ruminants. A total of 384 small ruminants were selected using a simple random sampling technique. Faecal samples were examined microscopically using Floatation and sedimentation techniques. A structured questionnaire was used to obtain information regarding the potential risk factors. Data were analysed using Stata/MP 19.5. A *p* value < 0.05 was considered statistically significant.

**Result:**

The overall prevalence of common gastrointestinal parasites was 73.4% in the study area. The prevalence of parasitic infection associated with the species was 79.0% and 63.57% in sheep and goats respectively. This study showed that sheep were more affected than goats, indicating a statistically significant difference (*p* = 0.025) between the species. The likelihood of gastrointestinal parasitic infection was increased by two‐fold (AOR = 2.16, CI = 1.36, 3.44, *p* = 0.001) in ovine than caprine. The prevalence of common gastrointestinal parasites was 77.2% in females and 68% in males, and there was no significant difference between sex (*χ* ^2^ = 12.38, *p* = 0.135). The prevalence of gastrointestinal parasites by age group was 72% in young animals, 59% in adults and 95.6% in older animals. There was a notable difference in infection rates across different age groups (*χ* ^2^ = 33.65, *p* = 0.006). The parasites found in ovine were *Trichostrongylus species* (10.24%), *Strongyloides species* (14.75%), *Haemonchus species* (11%), *Nematodirus species* (6.7%), *Fasciola species* (11.5%), *Eimeria species* (6.1%), *Trichuris species* (6.1%) and mixed infection (12.3%). The gastrointestinal parasite prevalence based on season was 88.5% in autumn, 63.9% in winter, 70.5% in spring and 70.6% in summer (*χ* ^2^ = 104.19, *p* = 0.000).

**Conclusion:**

This study found that common gastrointestinal parasites are the main economic challenges faced by small ruminants in the study area. The current study found that sheep had a higher rate of gastrointestinal parasites compared to goats. Therefore, proper strategic deworming of small ruminants should be carried out in the study area.

## 1. Introduction

Raising animals is a key source of income for small‐scale farmers in Ethiopia, and among them, small ruminants such as sheep and goats are the most valuable [1–3]. The country has about 42.9 million sheep and 52.5 million goats [1]. Livestock farming plays a key role in helping people in many developing countries improve their quality of life [2]. In sub‐Saharan African countries, livestock are important for the national economy and the way of life of people in rural areas [3]. Livestock offers power during droughts, supports crop growth, enriches the soil and supplies materials for industries [3, 4]. In the Horn of Africa, livestock contribute between 20% and 30% of the gross domestic product (GDP), and for individual farmers, up to 70% of their cash income comes from raising animals [4]. In Ethiopia, livestock makes up about 30%–35% of the country’s GDP and over 85% of the cash income for farmers. The livestock subsector also contributes approximately 12%–16% of the total GDP [5].

Small ruminants such as sheep and goats help support the livelihoods of millions of people, particularly those who are poor in developing countries. Small ruminants provide a significant source of income for farmers who do not own land in rural areas [6]. Infection by parasites in the gastrointestinal tract is a serious health issue that harms the productivity and breeding capabilities of sheep and goats in Ethiopia [6, 7]. Parasitic diseases cause significant economic losses because they lead to disease and deaths, and they also decrease the production of milk, meat, wool and slow down growth, resulting in losses of 20%–25% [7]. Among parasitic diseases, those that affect the gastrointestinal tract are the main cause of economic loss in the production and productivity of small ruminants [8]. Parasites in the gastrointestinal tract can seriously harm health and reduce the production and productivity of small ruminants [9]. Having gastrointestinal tract parasites can weaken an animal’s immune system, making them more likely to get other illnesses [10]. The issue is even worse in tropical countries due to the good environmental conditions that help gastrointestinal parasites spread easily [11].

Studies have been conducted in various regions of Ethiopia, showing that the prevalence of gastrointestinal nematode parasites in small ruminants varies. Iin Borena, Ethiopia, the prevalence was found in 74.7% [12]; in Southern Ethiopia, it was 98.9% [13]; in Bishoftu, Ethiopia, it was 86.7% [14]; around Gondar in Northern Ethiopia, it was 90.9% [15]; in Mendayo, Bunch, Southeast Ethiopia, it was 94.1% [15]; in Bako, Ethiopia, it was 36.7% [11]; in Asella, Ethiopia, it was 68.1% [16]; in Western Hararghe, it was 61.9% [14]; and in Western Oromia, the prevalence was 24.7% [17]. To date, no research has been conducted on the prevalence of gastrointestinal parasite infections in small ruminants in the Farta District of Northwest Ethiopia. This study aimed to estimate the prevalence of common gastrointestinal tract parasite infestations and their associated risk factors in small ruminants in the Farta District of Northwest Ethiopia.

## 2. Materials and Methods

### 2.1. Description of Study Areas

The study was conducted in the Farta District, South Gondar Zone in 2024. The Farta District is located in the South Gondar Zone of the Amhara Region of Ethiopia. It is located 595 km away from the capital city of Addis Ababa and at a latitude of 12°00′ 0.00″ N. Longitude: 38°00′ 0.00″ E. The annual rainfall amount of the area ranges from 1300 to 1850 mm, with a long‐term mean annual rainfall of 1500 mm. The altitude ranges from 856 to 3718 m above sea level, and the daily temperature ranges from 9 to 25 C. The animal population comprises 180,607 cattle, 80,158 goats, 130,653 sheep, 10,066 horses, 2000 mules, 20,765 donkeys, 800,950 poultry and 30,381 honeybee hives [18] (see Figure 1).

**FIGURE 1:**
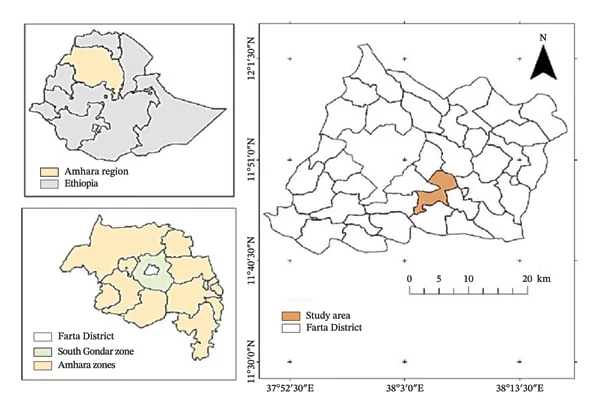
Map of Farta District, Northwest Ethiopia (created by QGIS Version 3.10.2 software).

### 2.2. Study Design

A cross‐sectional study was conducted between October 2024 and August 2025 to estimate the prevalence of gastrointestinal tract parasite infestations and their associated risk factors in small ruminants in the Farta District, Northwest Ethiopia.

### 2.3. Study Animals

This study focused on indigenous sheep and goat breeds raised by smallholder farmers in the study area. The study used animals from the Farta and Washera breeds, which were managed with an extensive system. Some farmers would keep their animals together on shared pastures during the day but split them up at night, placing each in their own space with a bit more feed. Others let their animals eat on the community land throughout the whole year without giving them extra feed.

### 2.4. Sampling Techniques and Sample Size Determination

Multistage sampling was used to choose the peasant associations, and the study animals were picked using simple random methods. The required sample size for this study was estimated by considering a 50% gastrointestinal tract parasite infestation in the study area. The sample size was calculated using the Thrusfield formula [19], and the analysis was done with an absolute precision of 5% and a 95% confidence level:

(1) N=Z2P1−Pd2,

where *N* = sample size, *P* = 50% expected prevalence and *Z* = 1.96 with 5% desired absolute precision (d). Therefore, the estimated sample size was 384 in this study.

### 2.5. Study Methodology

#### 2.5.1. Sample Collection and Examination

Fresh faecal samples were gathered directly from each animal’s rectum throughout the study using disposable gloves and kept in universal bottles. The samples were then taken to the veterinary clinic laboratory in screw‐cap bottles and stored in 10% formalin [20]. While collecting samples, information such as the animal’s breed, gender, age, the owner’s name and the peasant association were also recorded. Age was determined by examining the animal’s teeth, and the animals were divided into three groups: those young (< 5 years), those between 5 and 10 years (adult) and those older than 10 years (old). A total of 384 faecal samples were collected from sheep and goats that were chosen at random. Faecal smears, sedimentation, flotation or the Berman technique were used to find nematode eggs and larvae, following standard procedures [19].

The types of eggs were identified based on their appearance under the microscope. Microscopic examination was done at 10x and 40x magnifications to identify the types of gastrointestinal tract parasites based on the colour, shape and size of the eggs [19, 21–23].

One of the flotation fluids used was saturated sodium chloride. Trematode eggs were found using the sedimentation method because they are heavier than nematodes and protozoans, so they sink to the bottom instead of floating [24]. Positive faecal samples were grown in a laboratory to separate larvae and identify the main types of stomach and intestinal worms that affect small ruminants [21, 23].

### 2.6. Data Management and Analysis

The collected data were input into Microsoft Excel spreadsheets, accurately coded and then analysed using Stata/MP 19.5. Descriptive statistics were used to determine the prevalence of common gastrointestinal tract parasite infections in small ruminants. The association between parasite infection and risk factors like species, age, sex and season was calculated using the Chi‐square (*χ* ^2^) test) A 95% confidence level and 5% precision were applied. Univariable and multivariable logistic regression analysis was applied to assess the association between the outcome variable and the potential risk factors. Regression coefficients were used to estimate odds ratios for each risk factor variable. Odds ratios (ORs) with 95% confidence intervals (CIs) were used to evaluate the level of association of the prevalence and potential risk factor variables. In all analyses, a 95% CI and *p* value of less than 0.05 were considered significant for statistical associations between the prevalence and associated potential risk factors.

## 3. Results

### 3.1. Prevalence and Association of the Potential Risk Factors With Gastrointestinal Parasite Infections

A total of 384 faecal samples from small ruminants were examined. The overall prevalence of gastrointestinal parasite infection in both sheep and goats was 73.4%. The prevalence of gastrointestinal tract parasites associated with the species was 79.0% and 63.57% in sheep and goats respectively. This study showed that sheep were more affected than goats, indicating a statistically significant difference (*χ* ^2^ = 17.50, *p* = 0.025) between the species. The prevalence of gastrointestinal tract parasites related to sex was 77.2% and 68% in females and males respectively. There was no statistically significant difference between sex (*χ* ^2^ = 12.38, *p* = 0.135) as explained in Table 1.

**TABLE 1:** The Prevalence of common gastrointestinal tract parasites and their associated risk factors.

Independent variables	No. of animals examined	No. of overall positive (%)	The outcome variable (%)									
			A	B	C	D	E	F	G	M	Pearson *χ* ^2^	*p* value (pr)
Species											17.5	0.025^∗^
Ovine	244	193 (79.0%)	25 (10.24%)	36 (14.75%)	27 (11.0%)	17 (6.7%)	28 (11.5%)	15 (6.1%)	15 (6.1%)	30 (12.3%)		
Caprine	140	89 (63.57%)	19 (13.6%)	12 (8.4%)	8 (5.7%)	11 (7.8%)	15 (10.7%)	6 (4.3%)	4 (2.8%)	14 (10.0%)		
Sex											12.38	0.135
Female	224	173 (77.2%)	23 (10.3%)	34 (15.1%)	22 (9.8%)	13 (5.8%)	28 (12.5%)	16 (7.1%)	12 (5.3%)	25 (11%)		
Male	160	109 (68%)	21 (13%)	14 (8.7%)	13 (8.1%)	15 (9.4%)	15 (9.4%)	5 (3.1%)	7 (4.4%)	19 (11.9%)		
Age											33.65	0.006^∗^
Young	97	70 (72%)	5 (5%)	11 (11.3%)	12 (12.4%)	7 (7.2%)	7 (7.2%)	13 (13.4%)	5 (5%)	10 (10.3%)		
Adult	171	101 (59%)	20 (11.7%)	14 (8.1%)	5 (2.9%)	7 (4%)	22 (12.8%)	2 (1.1%)	7 (4%)	24 (14%)		
Old	116	111 (95.6%)	19 (16.3%)	23 (19.8%)	18 (15.5%)	14 (12%)	14 (12%)	6 (5.1%)	7 (6%)	10 (8.6%)		
Body condition											29.83	0.019^∗^
poor	151	106 (70.1%)	13 (8.6%)	11 (7.2%)	14 (9.2%)	12 (7.9%)	13 (8.6%)	16 (10.6%)	7 (4.6%)	20 (13.2%)		
Medium	149	109 (73.1%)	18 (12%)	24 (16.1%)	16 (10.7%)	12 (8.05%)	15 (10%)	2 (1.3%)	8 (5.4%)	14 (9.4%)		
Good	84	67 (79.7%)	13 (15.5%)	13 (15.4%)	5 (5.9%)	4 (4.7%)	15 (17.8%)	3 (3.5%)	4 (4.7%)	10 (11.9%)		
Season											104.1	0.000^∗^
Autumn	78	69 (88.5%)	11 (14.1%)	19 (24.3%)	7 (8.9%)	11 (14.1%)	2 (2.5%)	8 (10.2%)	7 (8.9%)	4 (5.1%)		
Spring	78	55 (70.5%)	14 (17.9%)	17 (21.7%)	9 (11.5%)	5 (6.4%)	0 (0.0%)	0 (0.0%)	3 (3.8%)	7 (8.9%)		
Summer	92	65 (70.6%)	6 (9.2%)	7 (7.6%)	9 (9.7%)	2 (2.1%)	20 (21.7%)	0 (0.0%)	2 (2.1%)	19 (20.6%)		
Winter	136	87 (63.9%)	13 (9.5%)	5 (3.7%)	10 (7.35%)	10 (7.35%)	21 (15.4%)	13 (9.5%)	1 (0.7%)	14 (10.2%)		

*Note:* A: *Trichostrongylus*; B: *Strongyloides*; C: *Haemonchus*; D: *Nematodirus*; E: *Fasciola spp*.; F: *Eimeria spp*.; G: *Trichuris*; M: Mixed infection. *χ* ^2^ = Pearson chi‐square.

^∗^Statistically significant.

The prevalence of gastrointestinal tract parasites related to age was 72%, 59% and 95.6% in young, adult and old animals respectively. The highest prevalence was 95.6% in old animals, while the lowest prevalence was 59% in adult animals. The prevalence of gastrointestinal tract parasites among the different age groups was statistically significant (*χ* ^2^ = 33.65, *p* = 0.006), as shown in Table 1.

The prevalence of gastrointestinal tract parasites associated with body condition was 70.1%, 73.1% and 79.7% in poor, medium and good body condition respectively. The prevalence of gastrointestinal tract parasites among the different body conditions was statistically significant (*χ* ^2^ = 29.83, *p* = 0.019). Therefore, species, age, body condition and season were considered potential risk factors for parasitic infections.

The prevalence of *Trichostrongylus, Strongyloides, Haemonchus, Nematodirus, Fasciola, Eimeria, Trichuris species* and mixed infection in sheep was 10.24%, 14.75%, 11%, 6.7%, 11.5%, 6.1%, 6.1% and 12.3% respectively. The prevalence of *Trichostrongylus*, *Strongyloides, Haemonchus, Nematodirus, Fasciola, Eimeria* and *Trichuris species* and mixed infection in goats was 13.6%, 8.4%, 5.7%, 7.8%, 10.7%, 4.3%, 2.8% and 10.0% respectively.

The prevalence of *Trichostrongylus, Strongyloides, Haemonchus, Nematodirus, Fasciola, Eimeria, Trichuris species* and mixed infection in females was 10.3%, 15.1%, 9.8%, 5.8%, 12.5%, 7.1%, 5.3% and 11% respectively. The prevalence of *Trichostrongylus, Strongyloides, Haemonchus, Nematodirus, Fasciola, Eimeria, Trichuris species* and *mixed infection* in males was 13%, 8.7%, 8.1%, 9.4%, 9.4%, 3.1%, 4.4% and 11.9% respectively.

The prevalence of the *Trichostrongylus , Strongyloides, Haemonchus, Nematodirus, Fasciola, Eimeria, Trichuris spp.* and mixed infection in young age were 5%, 11.3%, 12.4%, 7.2%, 7.2%, 6.18%, 13.4% 5% and 10.3%, respectively, while the prevalence of the *Trichostrongylus , Strongyloides, Haemonchus, Nematodirus, Fasciola, Eimeria* and *Trichuris spp.* and mixed infection in old age was 16.3%, 19.8%, 15.5%, 12%, 12%, 5.1%, 6% and 8.6% respectively (Table 1).

### 3.2. Prevalence of Gastrointestinal Tract Parasites in Different Peasant Associations

The prevalence of the common gastrointestinal tract parasites in sheep was 18.8%, 11%, 13.5%, 14.3%, 8.6% and 9.4% in the Abaregay, Selamko, Workie, Tsegure, Kanat and Magera peasant associations respectively. The highest prevalence of the common gastrointestinal tract parasite infection in sheep was 18.8% in Abaregay, while the lowest was 8.6% in Kanat. The prevalence of the common gastrointestinal tract parasite infection in both sheep and goats was 83.3%, 59%, 80.9%, 76.1%, 76.1% and 60.8% in Abaregay, Selamko, Workie, Tsegure, Kanat and Magera, respectively, as shown in Table 2. The prevalence of gastrointestinal tract parasites among the different peasant associations was statistically significant (*χ* ^2^ = 119.35 *p* = 0.000). The highest prevalence of the parasite was 83.3% in both sheep and goats in Abaregay, whereas the lowest prevalence was 59% in Selamko.

**TABLE 2:** The prevalence of common gastrointestinal tract parasites in sheep and goats in various peasant associations in Farta District, Northwest Ethiopia.

	Sheep		Goat		Both (sheep and goat)			(*χ* ^2^)	*p* value
	Total samples examined	Total positive (%)	Total samples examined	Total positive (%)	Total samples examined in both sheep and goat	Total positive in both sheep and goat	Prevalence both in sheep and goats (%)		
Peasant association								119.3520	0.000^∗^
Abaregay	53	46 (18.8%)	25	19 (13.5%)	78	65	83.3%		
Selamko	46	27 (11%)	20	12 (8.5%)	66	39	59%		
Workie	36	33 (13.5%)	27	18 (12.8%)	63	51	80.9%		
Tsegure	45	35 (14.3%)	18	13 (%)	63	48	76.1%		
Kanat	34	29 (8.6%)	29	19 (9.3%)	63	48	76.1%^∗^		
Magera	30	23 (9.4%)	21	8 (5.7%)	51	31	60.8%		

Overall prevalence	244	193 (79.0%)	140	89 (63.57%)	384	282	73.4%		

^∗^Statistically significant.

### 3.3. Prevalence of the Common Gastrointestinal Tract Parasites in Various Seasons

The prevalence of common gastrointestinal tract parasites in sheep was 97.7%, 69.7%, 75% and 81.9% in autumn, winter, spring and summer respectively. The prevalence of gastrointestinal tract parasites in goats was 75.7%, 66%, 61.5% and 48.3% in autumn, winter, spring and summer, respectively, as described in Table 3. The highest prevalence of gastrointestinal tract parasite infection in sheep was 97.7% in autumn, while the lowest was 69.7% in winter. The highest prevalence of gastrointestinal tract parasite infection in goats was 75.7% in autumn, while the lowest was 48.3% in summer. The prevalence of gastrointestinal tract parasites in both sheep and goats was 88.4%, 68.4%, 70.5% and 70.6% in autumn, winter, spring and summer, respectively. The prevalence of parasitic infection among the various seasons was statistically significant (*χ* ^2^ = 104.19, *p* = 0.000).

**TABLE 3:** Prevalence of gastrointestinal tract parasite infection in small ruminants based on season.

	Sheep		Goat		Both				
	Total samples examined	Total positive	Total samples examined	Total positive	Total samples examined in both	Total positive in both	Prevalence both in sheep and goats (%)	*χ* ^2^	*p* value
Season								104.1994	0.000^∗^
Autumn	45	44 (97.7%)	33	25 (75.7%)	78	69	(88.4%)		
Winter	86	60 (69.7%)	50	33 (66%)	136	93	(68.4%)		
Spring	52	39 (75%)	26	16 (61.5%)	78	55	(70.5%)		
Summer	61	50 (81.9%)	31	15 (48.3%)	92	65	(70.6%)		

Overall prevalence	244	193 (79.0%)	140	89 (63.5%)	384	282	(73.4%)		

^∗^Values varying significantly at *p* < 0.05.

### 3.4. Logistic Regression Analysis of the Potential Risk Factors

The potential risk factors for the common gastrointestinal tract parasites among small ruminants were identified using univariable and multivariable logistic regression analyses. A univariate analysis was applied with a 0.05 level of significance to select the required independent variables for multivariable analysis. SPP, sex and season were significantly associated with the parasitic infection, as described in Table 4. Accordingly, the likelihood of being infected with gastrointestinal parasites was increased two‐fold (AOR = 2.16, CI = 1.36, 3.44, *p* = 0.001) in ovine than caprine; the risk of being infected with parasites was increased two‐fold in female small ruminants (AOR = 1.58, CI = 1.00, 2.50, *p* = 0.047) than male small ruminants; The occurrence of parasitic infection was increased by 0.3 (AOR = 0.28, CI = 0.128, 0.617, *p* = 0.002), (AOR = 0.31, CI = 0.133, 0.728, *p* = 0.007) and (AOR = 0.314, CI = 0.137, 0.717, *p* = 0.006) in all summer, autumn and spring, respectively, than in the winter season.

**TABLE 4:** Univariate and multivariable logistic regression analysis of potential risk factors associated with parasite infection.

Independent variables	AOR	Std. err.	z	P > |z|	95% conf. Interval
*SPP*					
Ovine	2.168539	0.5114857	3.28	0.001^∗^	1.365832 3.443001
Caprine					

*Sex*					
Female	1.587156	0.369403	1.98	0.047^∗^	1.005788 2.504567
Male					

*Age*					
Young	1.184694	0.3410688	0.59	0.556	0.6738271 2.082878
Old	0.9701299	0.2969199	−0.10	0.921	0.5324875 1.767463
Adult					

*Body condition*					
Poor	1.15684	0.2968156	0.57	0.570	0.699643 1.912801
Medium	1.673141	0.5432044	1.59	0.113	0.8854829 3.16144
Good					

*Season*					
Summer	0.2821031	0.1127045	−3.17	0.002^∗^	0.1289258 0.6172709
Autumn	0.3119093	0.1349756	−2.69	0.007^∗^	0.1335609 0.7284123
Spring	0.3140097	0.1324903	−2.75	0.006^∗^	0.1373393 0.7179448
Winter					

^∗^Statistically significant.

## 4. Discussion

Parasites in the digestive system lead to financial losses in the raising of small ruminants in Ethiopia [25]. The current study found that 73.4% of small ruminants had gastrointestinal parasites, which is similar to the findings from Handiso’s study, which reported a 74.7% prevalence of parasites in the gastrointestinal tract of small ruminants in Borena, Ethiopia [23]. In the present study, the likelihood of being infected with gastrointestinal parasites was increased by two times (AOR = 2.16, CI = 1.36, 3.44, *p* = 0.001) in ovine than caprine. The current study also supports the findings from Kumsa’s report [24], which said that sheep are more likely to get infected with gastrointestinal worms because they eat grass in open fields and come into contact with worm eggs, whereas goats mainly eat leaves and twigs, which makes it less likely for them to get infected with these worms [26].

The prevalence of common gastrointestinal tract parasites related to sex was 77.2% and 68% in females and males, respectively. In this study, there was no meaningful difference between males and females (*χ* ^2^ = 12.38, *p* = 0.135), which matches the findings reported by Getachew [27]. The prevalence in the current study was lower compared to earlier research on small ruminants from various regions in Ethiopia. Earlier studies reported rates of 98.9% in Southern Ethiopia [28], 86.7% in Bishoftu [29], 90.9% in Gondar [30] and 94.1% in Mendayo [31].

The current result showed a higher rate compared to past studies, with percentages like 36.7% in Bako, Ethiopia [32], 68.1% in Asella, Ethiopia [33], 61.9% in Western Hararghe [34] and 24.7% in Western Oromia [35]. The variation in prevalence among findings was related to seasonal variation during the study period, the age of the animals, the stage of infestation and the treatment of animals [36]. Other factors, such as the management system, the time of year, the peasant association, the genetics of the host and the immunity of the animals, also played a role in how often gastrointestinal tract parasites were found [30, 37].

In this study, the percentage of sheep infected with *Trichostrongylus* , *Strongyloides, Haemonchus, Nematodirus, Fasciola, Eimeria*, *Trichuris species* and mixed infection was 10.24%, 14.75%, 11.0%, 6.7%, 11.5%, 6.1%, 6.1% and 12.3% respectively. The common worms, such as *trichostrongyles, Strongylus, Haemonchus, Nematodirus, Fasciola, Eimeria, Trichuris* and mixed infection, were found in 13.6%, 8.4%, 5.7%, 7.8%, 10.7%, 4.3%, 2.8% and 10.0% of goats, respectively, which match what Hailelul found [30]. In another study, it was found that 35% of small ruminants had Haemonchus and 29% had *Trichostrongylus species* [20, 38].

The *Trichostrongylus spp.* can better handle tough environmental conditions compared to *Haemonchus spp* [30]. About 12.82% of small ruminants are affected by *Nematodirus spp*. The current study agrees with Singh [39]. Mixed infections were also seen, such as *Trichuris, Fasciola, Strongyloides* and *Trichostrongylus spp.* The study found that *Trichostrongylus* parasites were the most common in small ruminants, which matches the results from Singh’s research [36]. This could be because the climate in the study area is good for the survival and growth of the infective larval stage of *Strongyles,* as shown in references [31, 38].

Age was seen as a risk factor, and 72% of the animals studied were young. The lowest rate was 59%, seen in adult animals. The findings of this study about how common gastrointestinal parasites are linked to age match what was reported by Yimer [34, 40]. The higher rate of intestinal parasite problems in young small ruminants could be because their immune system is not fully developed, they come into contact with more parasite eggs and larvae, they are less able to fight off infections, and they do not have much built‐up immunity from previous infections [30, 38]. This finding shows that age is a key factor in how likely small ruminants are to get infected with parasites in their digestive system [33].

The percentage of small ruminants infected with gastrointestinal parasites was 88.5% in autumn, 63.9% in winter, 70.5% in spring and 70.6% in summer. Unlike what is found now, Singh [43] reported that the most common gastrointestinal parasites were seen in autumn, then summer and then winter. The most common infection of stomach and intestinal worms in sheep happened during autumn, with 88.5% of the sheep and goats affected, and it was least common in winter, with only 63.9% of the sheep infected. During winter, the time animals spend grazing is less, and the early stages of parasites also become inactive, which helps keep infection rates low in the winter [20, 36]. This study showed that rainfall and temperature have a big impact on the spread of gastrointestinal parasites, according to Zvinorova [40].

## 5. Conclusion and Recommendations

Small ruminants provide a key source of income for farmers who do not own land in rural regions. Parasitic diseases affect the economy because they lead to a lot of illness, death and financial loss through reduced wool, milk and meat production. Gastrointestinal parasites, especially those that cause parasitic gastroenteritis, pose a major health risk and limit the production and productivity of small ruminants. The current research showed how common or widespread the gastrointestinal parasites are in small ruminants. Gastrointestinal worms were a significant economic problem for small ruminants in the study area. The study on faecal samples showed an overall prevalence of 73.4% in small ruminants in the study area. The study showed that sheep were more likely to have gastrointestinal parasites compared to goats. The rate at which parasites were found was greatly affected by age and the type of species. In light of the above conclusion, the following recommendations are put forward:

i. Strategic deworming of small ruminants for the control of common gastrointestinal tract parasites should be done seasonally.
ii. Public awareness should be created among animal owners about good husbandry systems by veterinarians.
iii. Further studies on economic losses and species identification of gastrointestinal parasites of small ruminants shall be conducted in the study area.

## Author Contributions

Balemual Abebaw was involved in suggesting the title, writing the report and preparing the manuscript, contributed to gathering data, writing the first draft, understanding the results and preparing the manuscript, and is also responsible for making sure the work is accurate and original.

## Funding

There is no funding for this research.

## Disclosure

The author has read and agreed to the published version of the manuscript.

## Ethics Statement

Before data collection, the Debre Tabor University Institutional Research Ethical Review Committee, with verification number IRERC/14/11/2024, provided ethical approval and clearance. Data collectors obtained informed consent from each study participant after explaining the study’s objectives and value to them.

## Conflicts of Interest

The author declares no conflicts of interest.

## Data Availability

The data that support the findings of this study are available from the corresponding author upon reasonable request.
